# Impaired novelty acquisition and synaptic plasticity in congenital hyperammonemia caused by hepatic glutamine synthetase deficiency

**DOI:** 10.1038/srep40190

**Published:** 2017-01-09

**Authors:** Aisa N. Chepkova, Olga A. Sergeeva, Boris Görg, Helmut L. Haas, Nikolaj Klöcker, Dieter Häussinger

**Affiliations:** 1 Institute of Neural and Sensory Physiology, Heinrich Heine University, D-40225 Düsseldorf, Germany; 2 Department of Gastroenterology, Hepatology and Infectious Diseases, Heinrich-Heine-University, D-40225 Düsseldorf, Germany; 3 Institute of Clinical Neurosciences and Medical Psychology, Heinrich Heine University, D-40225 Düsseldorf, Germany.

## Abstract

Genetic defects in ammonia metabolism can produce irreversible damage of the developing CNS causing an impairment of cognitive and motor functions. We investigated alterations in behavior, synaptic plasticity and gene expression in the hippocampus and dorsal striatum of transgenic mice with systemic hyperammonemia resulting from conditional knockout of hepatic glutamine synthetase (LGS-ko). These mice showed reduced exploratory activity and delayed habituation to a novel environment. Field potential recordings from LGS-ko brain slices revealed significantly reduced magnitude of electrically-induced long-term potentiation (LTP) in both CA3-CA1 hippocampal and corticostriatal synaptic transmission. Corticostriatal but not hippocampal slices from LGS-ko brains demonstrated also significant alterations in long-lasting effects evoked by pharmacological activation of glutamate receptors. Real-time RT-PCR revealed distinct patterns of dysregulated gene expression in the hippocampus and striatum of LGS-ko mice: LGS-ko hippocampus showed significantly modified expression of mRNAs for mGluR1, GluN2B subunit of NMDAR, and A1 adenosine receptors while altered expression of mRNAs for D1 dopamine receptors, the M1 cholinoreceptor and the acetylcholine-synthetizing enzyme choline-acetyltransferase was observed in LGS-ko striatum. Thus, inborn systemic hyperammonemia resulted in significant deficits in novelty acquisition and disturbed synaptic plasticity in corticostriatal and hippocampal pathways involved in learning and goal-directed behavior.

Hyperammonemia is a major factor in the pathogenesis of encephalopathies associated with acute and chronic liver failure. Hepatic encephalopathy (HE) presents a spectrum of neuropsychiatric abnormalities varying from minimal cognitive and motor disturbances to coma, and its clinical symptoms are often ameliorated by reducing systemic ammonia levels, provided that terminal brain edema has not yet developed^1^,^2^,^3^.

A low ammonia level in systemic circulation is largely secured by the activity of liver urea cylce enzymes converting ammonium ions to non-toxic urea. For efficient entry into the urea cycle through low-affinity carbamoylphosphate synthetase activity, locally high concentrations of ammonia are required, which are established by periportal glutaminase activity. Spillover of these high concentrations of hepatic ammonia into systemic circulation is normally prevented by a high-affinity uptake into perivenous hepatocytes and local glutamine synthetase (GS) activity. Thus, acinar compartmentalization of urea synthesis, glutamine hydrolysis, and glutamine synthesis protects from systemic increases in ammonia concentrations^4^,^5^. In the brain, astroglial GS is largely responsible for the removal of both, blood-derived and metabolically generated ammonia^6^,^7^.

Hyperammonemia may be caused by a number of inherited disorders including defects in urea cycle enzymes^8^. As the developing brain is more sensitive to hyperammonemia compared to the adult, high ammonia levels in pediatric patients often provoke irreversible brain damage with mental retardation, seizures, and cerebral palsy^9^,^10^. Several animal models of chronic hyperammonemia with and without liver failure have been established to unravel the molecular mechanisms of ammonia toxicity^11^,^12^,^13^,^14^. However, the vast majority of such models deal with the effects of acquired hyperammonemia on adult brain function reproducing many of the clinically observed symptoms in HE. Until recently, the only available animal model of congenital hyperammonemia was the sparse-fur (*spf*) mouse carrying a point mutation in the ornithine transcarbamoylase (OTC) gene^15^,^16^. *Spf* mice have blood ammonia levels that are 2–3fold higher than normal; they show cerebral atrophy, significant alterations in neurotransmitter systems, behavioral abnormalities, and a decreased life span^16^,^17^,^18^,^19^,^20^,^21^. As OTC is central for the mitochondrial portion of the urea cycle, it is not surprising that *spf* mice also show adaptive alterations in other mitochondrial enzyme activities and deficits in energy metabolism making a clear deduction of findings from mere hyperammonemia rather difficult. A novel mouse model of congenital hyperammonemia with liver-specific knockout of GS^22^ is used here and referred to as “LGS-ko”. Selective deletion of GS from perivenous hepatocytes in LGS-ko mouse affected neither urea cycle enzymes nor amino acid metabolism but caused a persistent, 3-fold increase in blood ammonia levels, associated with an oxidative stress response in the brain and behavioral abnormalities^22^. In contrast to liver specific GS knockout, congenital GS mutation in humans leads to severe cerebral malformations and neonatal death^23^. In the present study, we investigated exploratory behavior, synaptic plasticity, and plasticity-related gene expression in the hippocampus and striatum of mice lacking hepatic GS. We found, that congenital hyperammonemia caused specific deficits in novelty acquisition and synaptic plasticity in corticostriatal and hippocampal pathways that are implicated in learning behavior and goal-directed locomotion.

## Results

### Mice lacking hepatic GS show decreased exploratory activity

**Figure 1:**
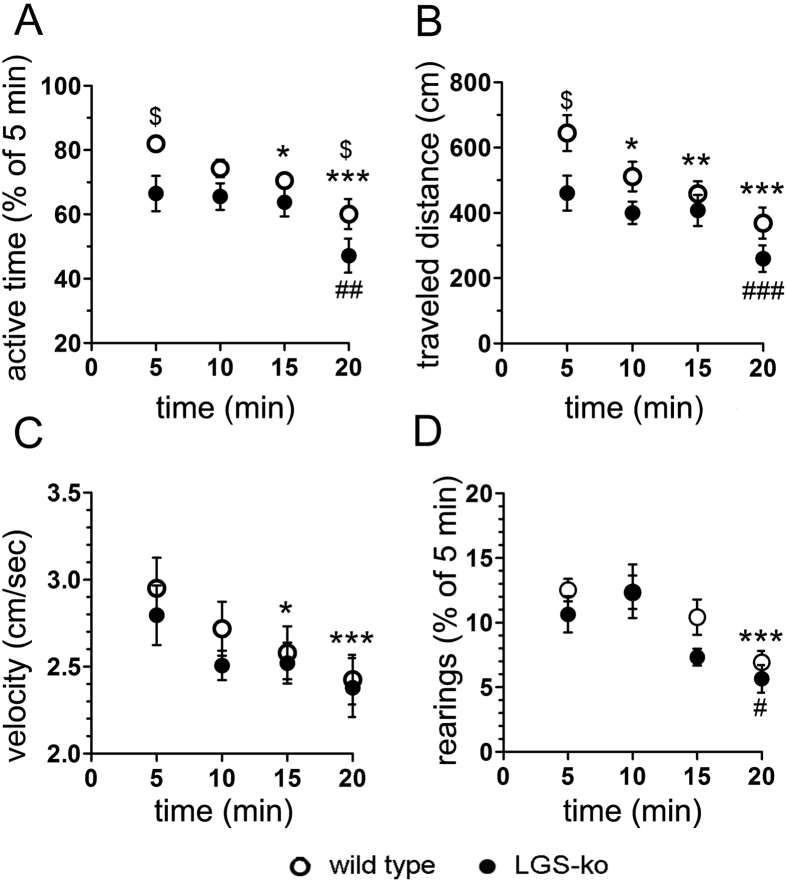
Exploratory activity is reduced in congenital hyperammonemia. Open field activity of mice lacking hepatic GS (filled circles, n = 12) and their wild type littermates (open circles, n = 11) recorded in a modified SmartCage^TM^ system for an observation period of 20 min. Behavioral parameters were averaged throughout time blocks of 5 min each. (**A**) active time. (**B**) traveled distance. (**C**) speed of horizontal movements (velocity). (**D**) vertical activity (rearings). The dollar sign indicates an overall significant difference in respective parameters (^$^p < 0.05, t-test). Asterisks and hash signs mark significant differences in recorded parameters in indicated time blocks from their initial values in wild type and LGS-ko animals, respectively (repeated measures ANOVA, *,^#^p < 0.05, **,^##^p < 0.01, ***,^###^p < 0.001).

To assess exploratory activity, mice were placed into SmartCages^TM^ and both horizontal (ambulances) and vertical (rearings) movements were recorded for a period of 20 min. As can be derived from Fig. 1, LGS-ko animals (n = 12) showed significantly less active time and less traveled distance compared to wt animals (n = 11), both initially (p < 0.05, t-test) and over the whole period of observation (p < 0.05 for genotype and time, two-way ANOVA), whereas the parameters “movement velocity” and “rearings”, the latter of which represents another component of exploratory behavior, did not differ between genotypes. Total active time was reduced from 14.3 ± 0.4 min in wt to 12.1 ± 0.7 min in LGS-ko (p = 0.0189, t-test; Fig. 1A) and traveled distance was decreased from 1985 ± 146 cm in wt to 1529 ± 135 cm in LGS-ko animals (p = 0.0025, t-test; Fig. 1B).

Two approaches were used to evaluate within-session habituation to novel environment: (i) quantification of the relative decrease in respective parameters at the end of the observation period; (ii) repeated measures ANOVA to assess putative alterations in consecutive time blocks. The first approach yielded no significant difference in habituation between genotypes. Thus, active time decreased to 60 ± 8% and 68 ± 12% of initial values in WT and LGS KO, respectively, traveled distance was reduced to 74 ± 6% and 74 ± 8%, movement velocity slowed to 87 ± 7% and 84 ± 5%, and rearings declined to 56 ± 7% and 79 ± 32%, respectively. Repeated measures ANOVA, however, indicated differences in the dynamics of within-session habituation. Active time was significantly reduced in LGS KO mice only within the last time block (2.36 ± 0.26 min versus 3.32 ± 0.28 min, initially; p < 0.01), whereas in WT mice, this parameter significantly decreased already within the third block (3.52 ± 0.09 min versus 4.1 ± 0.1 min, initially; p < 0.05) and continued to decrease further within the last time block of the observation period (3.01 ± 0.23 min, p < 0.001). Similarly, traveled distance significantly decreased in LGS KO mice only within the last 5 min, whereas in WT mice it significantly dropped already within the second time block (p < 0.05) and continuously decreased afterwards (p < 0.01 and p < 0.001 when comparing the third and forth time block with the initial one, respectively). In addition, LGS KO mice did not show a significant reduction of their movement velocity within the total period of observation, whereas WT mice did so within the last two time blocks (p < 0.05 and p < 0.001 compared to the initial block, respectively). Independent of genotype, rearings were reduced only in the last time block of observation when compared to the initial one (0.28 ± 0.05 min versus 0.53 ± 0.07 min (p < 0.05) and 0.35 ± 0.04 min versus 0.63 ± 0.04, (p < 0.001) for LGS KO and WT mice, respectively).

In summary, mice lacking hepatic GS showed decreased exploratory activity when placed in a novel environment. Moreover, habituation to such novel environment starts with a delay in these animals. Considering that changes in behavioral parameters may be associated with neurochemical alterations in hippocampal and corticostriatal neural circuits and corresponding alterations in neuroplasticity we studied synaptic plasticity and gene expression of selected receptors and enzymes involved in hippocampal and corticostriatal neurotransmission in LGS-ko mice.

### Selective impairments of hippocampal plasticity in mice lacking hepatic GS

**Figure 2:**
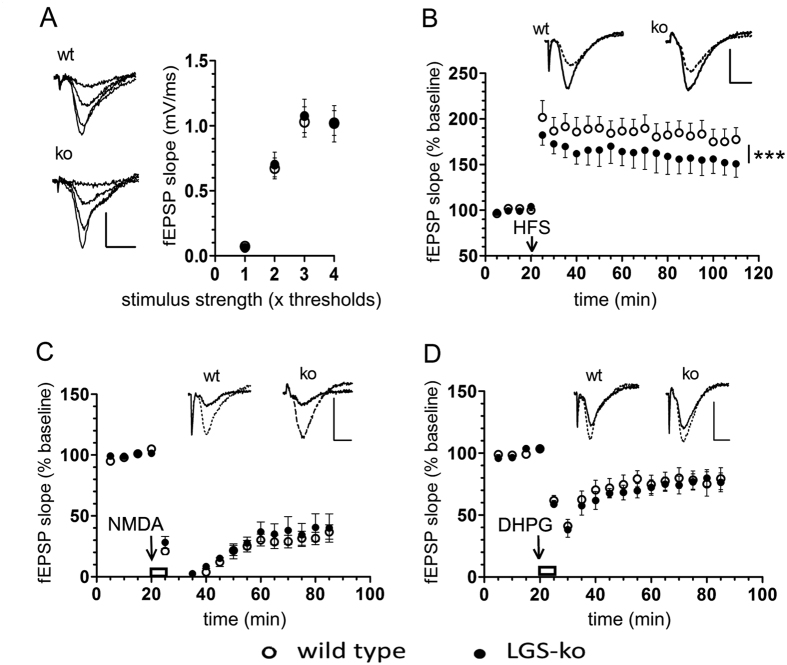
Hippocampal LTP is selectively constrained in congenital hyperammonemia. (**A**) Basal CA3 – CA1 synaptic transmission is not affected by hepatic GS deficiency. Representative examples of CA1 fEPSPs elicited by stimulation of Schaffer collateral/commissural input with increasing stimulus strength (average of 3 responses to a given stimulus intensity) and average stimulus-response relations obtained in slices from wild type mice (wt, n = 38, open circles) and from LGS-ko mice (ko, n =  29, filled circles). (**B**) Long-term potentiation (LTP) induced by high frequency electrical stimulation (HFS) is impaired in LGS-ko mice. Top: representative examples of CA1 fEPSPs averaged through 5 min before (dotted line) and 85–90 min after HFS (solid line) in wt and ko slices. Below: average changes in CA1 fEPSP slopes (***p < 0.0001, two-way ANOVA). (**C**) Long-term depression (LTD) of CA3 – CA1 synaptic transmission induced by pharmacological stimulation of the NMDA subtype glutamate receptors. Upper traces - representative examples of CA1 fEPSPs (average of 10 responses) in slice preparations from wt and LGS-ko animals 5 min before application of 20 μM NMDA (dotted line) and during the last 5 min of the washout period (solid line). Plot shows the average time courses of changes in CA1 fEPSP slopes induced by 5 min perfusion of hippocampal slices with NMDA (open bar). (**D**) LTD of CA3 – CA1 synaptic transmission induced by pharmacological stimulation of the mGluRI subtype of glutamate receptors. Upper traces - representative examples of CA1 fEPSPs (average of 10 responses) in slice preparations from wt and LGS-ko animals 5 min before (dotted line) application of 100 μM S-3,5-dihydroxyphenylglycine (DHPG) and during the last 5 min of the wash out period (solid line). Plot shows the average time courses of changes in CA1 fEPSP slopes after 5 min slice perfusion with DHPG (open bar). Scale bars in (**A**–**D**): 1 mV, 5 msec.

In a first set of experiments, we characterized basal neurotransmission in acute hippocampal slices of wt and LGS-ko mice by quantifying fEPSPs in the CA1 area stimulating the Schaffer collateral and commissural input with increasing stimulus strength. As shown in Fig. 2A, there was no effect of genotype on the stimulus-response relation (two-way ANOVA). In order to detect possible alterations in hippocampal synaptic plasticity by hepatic GS deficiency, long-term potentiation (LTP) in CA1 pyramidal neurons induced by high frequency stimulation (HFS) of Schaffer collateral-commissural input and long-term depression (LTD) in CA1 pyramidal neurons induced by agonists at N-methyl-D-aspartate (NMDA) receptors (NMDARs) and group I metabotropic glutamate receptors (mGluRs) were characterized. These experimental approaches classified as “chemical LTD” share some molecular properties with LTD induced by electrical stimulation of selected pathways^24^.

HFS of afferent input of CA1 pyramidal neurons resulted in either sustained (preferentially) or only transient potentiation of neurotransmission decaying during the recording period. The occurrence of sustained potentiation was not affected by genotype: in 8 out of 13 LGS-ko slices, sustained potentiation was observed as compared to 7 out of 12 slices of wt hippocampus (p = 1, 0 Fisher’s exact test). However, there was a significant influence of genotype on the magnitude of sustained LTP. As shown in Fig. 2B, LTP magnitude in LGS-ko hippocampus was significantly below wt values throughout the whole 90 min observation period (p  < 0.001 two-way ANOVA).

Short exposure (5 min) of wt hippocampal slices to NMDA (20 μM) or to the agonist of group 1 mGluRs DHPG (100 μM) resulted in chemically- induced LTD of CA3 – CA1 neurotransmission (Fig. 2C and D). In contrast to the observed effects on HFS-induced LTP, neither the acute responses to NMDA and DHPG nor the occurrence or magnitudes of chemically-induced LTD were altered in hippocampal slices from LGS-ko compared to wt animals.

### Complex impairment of corticostriatal plasticity in mice lacking hepatic GS

**Figure 3:**
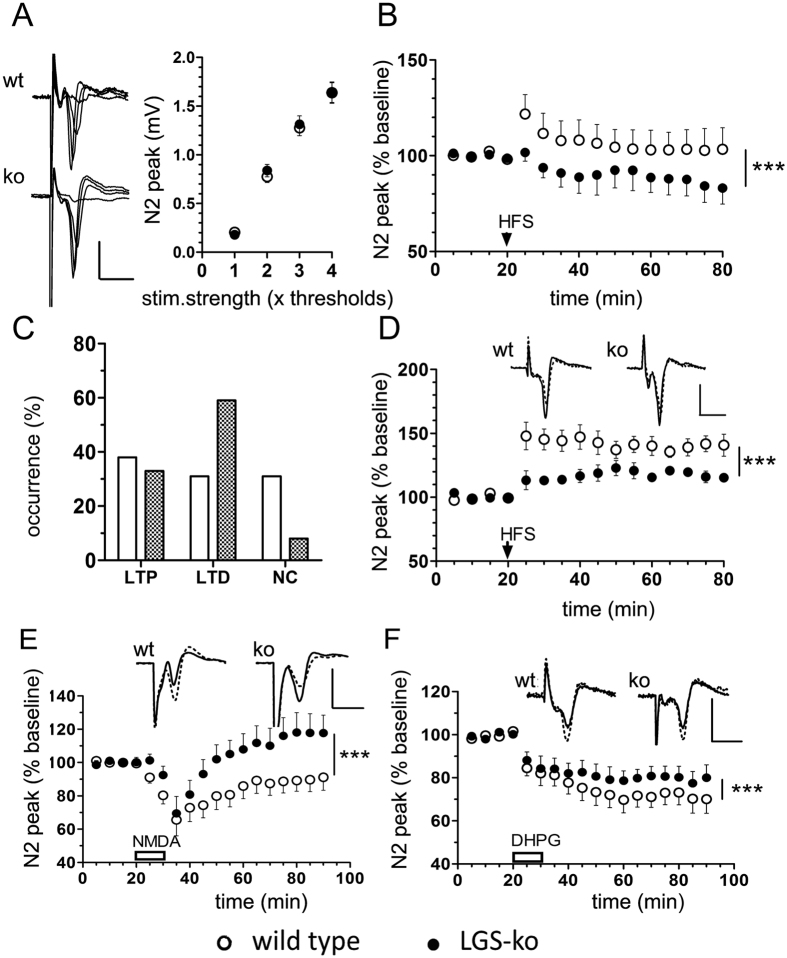
Corticostriatal plasticity is severely disturbed in congenital hyperammonemia. (**A**) Striatal field potentials (SFP) elicited by stimulation of subcortical white matter with increasing stimulus strength (average of 3 responses to a given stimulus intensity) and average stimulus-response relations obtained in slice preparations from wild type (wt, n = 46) and LGS-ko (ko, n = 39) mice. (**B**) Changes of SFP in slices from wt (n = 13) and LGS-ko (n = 12) mice in response to high frequency electrical stimulation (HFS) (***p < 0.0001, two-way ANOVA). (**C**) Occurrence of long-term potentiation (LTP), long-term depression (LTD), or no changes (NC) in SFP after HFS in slices from wt (open bars) and ko (filled bars) mice (no significant difference between genotypes, p = 0.2601, Chi-square test). (**D**) Impairment of corticostriatal HFS-induced LTP in LGS-ko mice. Top: averages of 10 SFP recorded for 5 min before (dotted line) and 55 – 60 min after HFS (solid line). Below: LTP time course in wt (n = 5) and ko (n = 4) mouse slices (***p < 0.0001, two-way ANOVA). (**E**) Long-term effects of short exposure to NMDA on corticostriatal neurotransmission depend upon genotype. Top: averages of 10 SFP in slices from wt and LGS-ko mice recorded for 5 min before (dotted line) and 55–60 min after (solid line) 10-min perfusion with 20 μM NMDA (open bar). Below: average time courses of changes in SFP in slices from wt (n = 13) and LGS-ko (n = 13) mice. (***p < 0.0001, two-way ANOVA). Note that NMDA application yields largely corticostriatal LTD in slices from wt mice and LTP in mice lacking hepatic GS. (**F**) LGS-ko mice show decreased LTD after pharmacological stimulation of the mGluRI subtype of glutamate receptors. Top: averages of 10 SFP in slices from wt and LGS-ko animals recorded for 5 min before (dotted line) and 55–60 min after (solid line) 10-min perfusion with 100 μM DHPG. Below: average time courses of changes in SFP in the two genotypes (***p < 0.0001, two-way ANOVA). Scale bars in (**A,D–F)**: 1 mV, 5 msec.

To assess the properties of basal neurotransmission in the corticostriatal pathway we analyzed changes in the amplitude of postsynaptic peak response (N_2_ peak) with increasing intensity of the cortical stimulus. Just as observed for hippocampal slices, genotype did not affect the stimulus-response relation in striatal slices when comparing LGS-ko with wt mice (Fig. 3A; two-way ANOVA). Next, we investigated whether synaptic plasticity was altered in corticostriatal neurotransmission in LGS-ko mice by characterizing the long-term effects of HFS of cortical input on striatal neurons or by short exposure of slices to NMDA or the group 1 mGluR agonist DHPG.

As shown in Fig. 3B, long-term effects of HFS on the corticostriatal field potential response depended upon genotype (p < 0.0001, two-way ANOVA). The mean response amplitude at 45–60 min post-HFS constituted 84 ± 4% of baseline in LGS-ko striatum, which is significantly lower than the mean wt value of 103 ± 5% of baseline (p = 0.0026, *t*-test). As can be derived from Fig. 3C, different occurrence of LTP and LTD in corticostriatal neurotransmission after HFS partially accounts for the genotype-dependent reduction in overall response. Whereas in the striatum of wt animals, cortical HFS induced LTP, LTD or no long-term changes (NC) with approximately equal probability, it induced largely LTD in the striatum of LGS-ko animals. If, however, LTP was induced by HFS in LGS-ko striatum, the magnitudes were significantly lower than in the striatum of wt animals (Fig. 3D). Average LTP magnitude at 45–60 min post-HFS constituted only 118 ± 2% of baseline in LGS-ko striatum versus 139 ± 3% of baseline in wt (p < 0.0001, *t*-test). The two genotypes did not significantly differ in the magnitudes of HFS-induced LTD. At 45–60 min post-HFS, average responses were 67 ± 3% and 60 ± 5% of baseline for LGS-ko and wt striatum, respectively (p = 0.1755, *t*-test, data not shown).

Unlike corticostriatal LTD elicited by electrical stimulation, chemically-induced depression did significantly differ between genotypes (p < 0.0001, two-way ANOVA, Fig. 3E/F). Bath application of NMDA (20 μM) for 10 min induced initial depression of field responses of equal extent in both wt and LGS-ko striatum (66 ± 9% and 69 ± 10% of baseline in wt and LGS-ko, respectively). In wt slices, this was followed usually by LTD (72 ± 3% of baseline, n = 7); occasionally, LTP developed (126 ± 6% of baseline, n = 2) and some slices did not show any long-term changes in field responses after NMDA stimulation (n = 4). In clear contrast, the majority of LGS-ko striatal slices developed LTP after initial depression by NMDA perfusion (141 ± 5% of baseline, n = 8), whereas LTD (69 ± 2% of baseline, n = 3) or no long-term change (n = 2) were the less frequent responses. Thus, summing up all responses, NMDA-induced LTD in wt striatum was reversed into LTP in LGS-ko genotype (90 ± 3% of baseline and 117 ± 5% of baseline for wt and LGS-ko, respectively, n = 13, p < 0.0001, t-test). Also, the distribution of NMDA-induced LTD versus LTP responses was significantly different between genotypes (p = 0.0348, one-tailed Fisher’s exact test), underlining that NMDA induced opposite long-term effects on corticostriatal neurotransmission.

Bath application of DHPG (100 μM) equally reduced the amplitude of corticostriatal field responses in wt and LGS-ko mice by the end of the 10 min perfusion period (81 ± 5% and 84 ± 5% and of baseline, respectively, n = 14). However, whereas in LGS-ko preparations, DHPG-induced depression remained at approximately the same level (80 ± 3% of baseline at 45–60 min post-DHPG), it became more pronounced in wt striatum (72 ± 3% of baseline at 45–60 min post-DHPG, p = 0.0477, *t*-test). Thus, corticostriatal slices from LGS-ko mice showed significantly less pronounced DHPG-induced LTD (DHPG-LTD).

### NMDAR potentiation and D2R inhibition level genotype-dependent differences in corticostriatal plasticity

**Figure 4:**
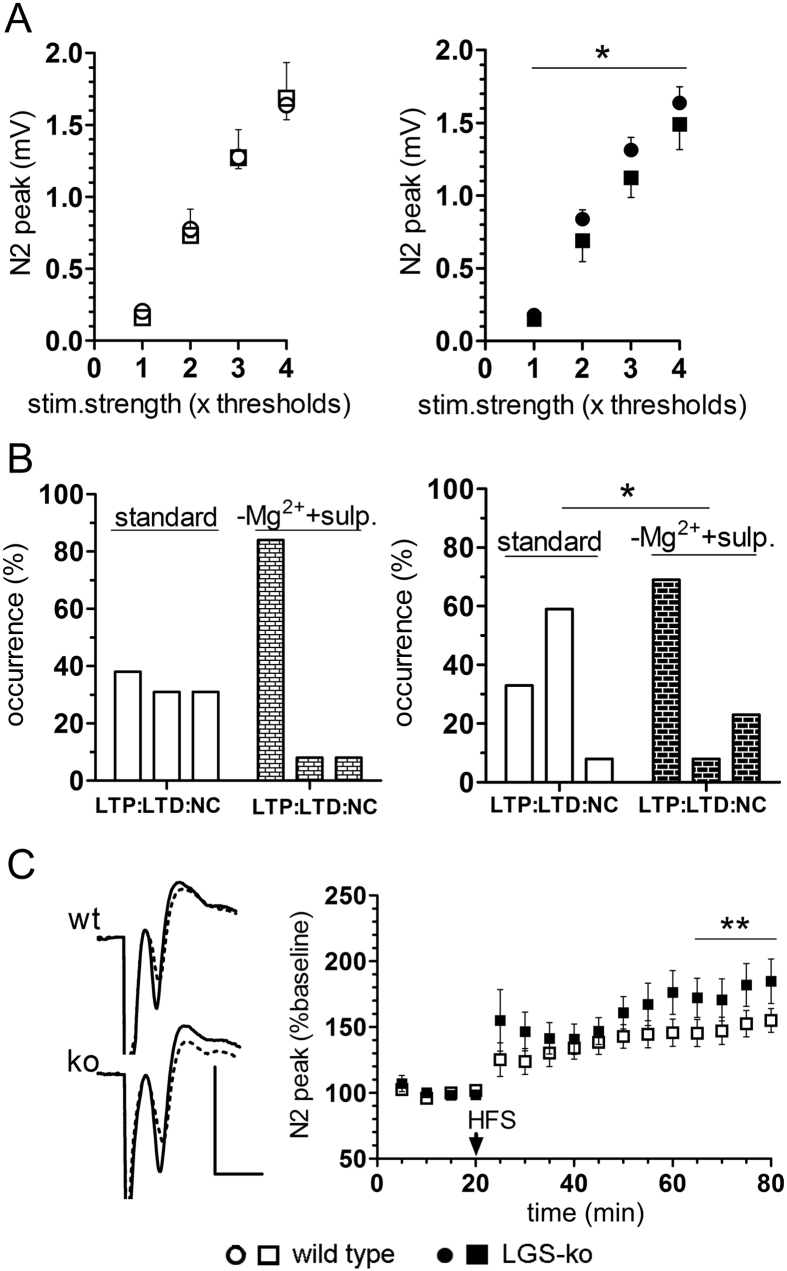
NMDAR activation and D2R inhibition unmask genotype-dependent differences in corticostriatal basal neurotransmission but balance them in plasticity. (**A**) Stimulus-response relations quantified from striatal field potentials elicited by stimulation of subcortical white matter in slice preparations from wild type (wt, left plots, open symbols) and mice deficient for hepatic GS (LGS-ko, right plots, filled symbols) in standard medium (circles) and modified Mg^2+^-free medium (squares) laced with the D2 receptor antagonist sulpiride (10 μM). The asterisk marks the significant reduction in corticostriatal responsiveness in LGS-ko mice by NMDAR activation and D2R inhibition (p = 0.0335, two-way ANOVA). (**B**) Occurrence of long-term potentiation (LTP), depression (LTD), and no change (NC) recorded from wt mice (left diagrams) and LGS-ko (ko, right diagrams) preparations perfused with standard (open bars) and modified medium (filled bars). Significant difference is indicated with asterisk (p = 0.0248 versus p = 0.073 in wt mice, Chi-square test). (**C**) NMDAR activation and D2R inhibition increases LTP magnitude in LGS-ko over wt mice. Representative examples of HFS-induced LTP of corticostriatal field responses (average of 10 responses) in wt and ko slice preparations, recorded for 5 min before (dotted line) and 55–60 min after HFS (solid line), and average time courses of changes in corticostriatal field responses in striatal slices from wt (n = 10) and LGS-ko (n = 9) mice showing LTP after subcortical HFS (arrow) in modified medium. Asterisks mark the significant difference between genotypes (p = 0.0038, t-test). Scale bars: 1 mV, 5 msec.

The experiments on corticostriatal neurotransmission and its plasticity in slices continuously perfused with Mg^2+^-free solution (to remove the voltage-dependent block of NMDARs) containing the D2R antagonist sulpiride (10 μM) revealed a small but significant decrease in the stimulus-response relation in striatal slices from LGS-ko mice (p = 0.0394, two-way ANOVA), whereas no such changes could be observed in wt striatum (Fig. 4A). As expected, NMDAR potentiation and D2R inhibition considerably increased the occurrence of corticostriatal LTP after HFS in both genotypes (Fig. 4B), advancing the induction of LTP to the main response in the majority of tested slices (10 out of 12 and in 9 out of 13 slices of wt and LGS-ko animals, respectively).

Statistical analysis of the alterations in the distribution of HFS – response categories LTP, LTD, and no long-term changes (NC) showed a significant influence of NMDAR potentiation and D2R inhibition in LGS-ko but not in wt striatal slices (p = 0.0248 and p = 0.073 in LGS-ko and wt, respectively, Chi-Square test). Finally, the magnitude of corticostriatal LTP induced by HFS was much more potentiated by Mg^2+^-free solution containing sulpiride in slices of LGS-ko than of wt mice. At 45–60 min after HFS, the average magnitude of LTP was 177 ± 8% in LGS-ko and 150 ± 5% in wt striatum (p = 0.0038, t-test). Compared to LTP induced by HFS under standard conditions (Fig. 3D), it was hence significantly potentiated in LGS-ko striatum (by a factor of 1.5, p < 0.0001, t-test) but only modestly increased in wt striatum (by a factor of 1.1, p = 0.0662, t-test). In summary, enhancing NMDAR mediated transmission and inhibiting D2R function exerted more pronounced effects on both the stimulus-response relation and HFS-induced plasticity in LGS-ko compared to wt striatum.

### Genotype-specific transcription of the selected genes

What are the molecular substrates underlying the behavioral and electrophysiological changes observed in LGS-ko mice? We performed a between-genotypes comparison of mRNA levels encoding for the selected neurotransmitter receptors, enzymes and structural proteins involved in synaptic plasticity. Using first PCR array “Mouse Nitric Oxide Signaling Pathway” (PAMM-062Z, SA Bioscience) as described in Chepkova *et al*.^25^ we detected a 2-fold upregulation of beta-actin expression in striatal tissue of LGS-ko mice with no change in any other gene transcripts when reactions were normalized on beta-2-microglobulin (B2m), glyceraldehyde-3-phosphate dehydrogenase (GAPDH) and glucuronidase beta (Gusb). Conventional real-time PCR confirmed significant up-regulation of beta-actin in LGS-ko striatum but not in the CA1 region of the hippocampus (Table 1). Other housekeeping genes (SDHA, B2m, Hsp90) did not change their activities in LGS-ko striatum, whereas heat shock protein 90 (Hsp90) and beta-2-microglobulin (B2m) were significantly up-regulated in the CA1 region of LGS-ko hippocampus (Table 1).

In the hippocampus of LGS-ko animals, we found significant up-regulation of mGluR1 mRNA expression, whereas mRNAs encoding the NMDAR subunit GluN2B and the adenosine receptor A1 were down-regulated. In the striatum of LGS-ko mice, mRNA expression of the D1 dopamine receptor (D1R) and the type 1 muscarinic cholinergic receptor (M1R) was up-regulated, accompanied by a down-regulation of choline-acetyl-transferase (ChAT). Thus, the LGS-ko genotype caused distinct patterns of dysregulated gene expression involved in synaptic plasticity in both the hippocampus and the striatum.

## Discussion

We demonstrate herein the alterations in exploratory behavior, synaptic plasticity and gene expression in the brain of mice with congenital hyperammonemia caused by deletion of hepatic GS. This experimental model is unique and characterized by a chronic hyperammonemia without impairment of liver function, blood flow and structure^22^. These mice were characterized by lowered exploratory activity in a novel environment, delayed within-trial habituation, and by reduced magnitude of electrically-induced hippocampal and corticostriatal LTP. Pharmacological activation of NMDAR and group I mGluR to induce chemical LTD revealed significant alterations in the long-term effects of both NMDA and DHPG in the LGS-ko striatum but not the hippocampus. These two structures showed also distinct patterns of alterations in gene expression.

In our behavioral tests, LGS-ko mice displayed reduced exploratory activity compared to their wild type littermates, when placed in a novel environment. As exploratory activity correlates inversely with anxiety and fear^26^, the behavioral phenotype of LGS-ko mice could be interpreted as enhanced fear. However, as previous O-maze testing has demonstrated decreased rather than increased fear in these mice, and even hyperactivity was reported in a familiar environment^22^, the behavior we observed might alternatively indicate deficient processing of novel information and impaired exploratory learning. This view is strongly supported by the delayed habituation in our experiments. Similar abnormalities in exploratory behavior and habituation have previously been described in an animal model of hepatic encephalopathy (HE), in which portacaval shunting caused chronic hyperammonemia in adult rats^27^,^28^.

It has been shown that assessing novel information about the environment involves parallel processing in both hippocampal and striatal neural circuits^29^, with the level of exploratory activity correlating with the striatal release of the neurotransmitters glutamate, dopamine, and acetylcholine^30^,^31^,^32^. The significant alterations in the expression of the M1R, D1R, and ChAT genes we found in the striatum of LGS-ko mice might therefore imply imbalances between cholinergic and dopaminergic neurotransmission, which could hamper proper function of cortico-basal ganglia circuits involved in linking novel information processing with motor activity.

The present study reveals a significant reduction in the magnitude of both hippocampal and corticostriatal LTP in LGS-ko mice when induced by HFS of synaptic input. Our results are in good agreement with previously reported rat models of chronic hyperammonemia and HE, in which animals either received an ammonium-rich diet^33^,^34^ or portacaval shunting^28^,^35^,^36^.

In both neuronal circuitries considered here, HFS-induced LTP critically depends on activation of NMDARs^37^,^38^,^39^, triggering activation of Ca^2+−^dependent enzymes and a consequent recruitment of AMPARs in the postsynaptic density (PSD) increasing synaptic strength^40^,^41^. According to previous studies on rat models of chronic hyperammonemia and HE^34^,^35^,^42^, the deficit in hippocampal LTP is associated with an altered function of the NMDAR-nitric oxide (NO)- cGMP- protein kinase G (PKG)- cGMP-degrading phosphodiesterase (PDE5) pathway, which is involved in LTP induction and maintenance, and with the dysfunction of Ca^2+−^calmodulin-dependent proteinkinase II (CaMKII) leading to impaired phosphorylation and trafficking of the GluA1 subunit of AMPAR. In an *in vitro* model of chronic hyperammonemia, we have shown that the lack of chemical NMDAR-dependent LTP in dissociated hippocampal neurons (DIV14–16) by high ammonia load was associated with the depletion of the extrasynaptic AMPAR reserve pool necessary for recruitment in LTP^43^. In the present mouse model, however, we did not find any changes in the mRNA expression of the AMPAR pore-lining subunits GluA1–4, neither in the hippocampus nor the striatum. Profound differences in the two model systems including the time course of the ammonia load, the methodological approach of detecting GluA expression selectively in juvenile neurons versus dissected whole CA1 region of adult mouse and other reasons might account for the discrepancy. We did, however, detect a significant down-regulation of the NMDAR subunit GluN2B in hippocampal tissue, which could explain the reduction of HFS-LTP in the hippocampus of LGS-ko mice^44^,^45^. Functional consequences of transcriptional downregulation of adenosin A1 receptors in the CA1 region merit further analysis. However, synaptic transmission in CA1 is controlled by presynaptic A1R expressed by CA3 pyramidal cells and deletion of A1R in CA1 does not influence synaptic responses^46^.

The induction of corticostriatal LTP requires co-activation of the dopamine D1R and the acetylcholine M1R^47^,^48^. Given the transcriptional up-regulation of D1R and M1R in LGS-ko striatum, enhancement rather than suppression of corticostriatal LTP induced by HFS would have been expected in these mice. However, receptor up-regulation may reflect compensation for a deficient synthesis or release of corresponding neurotransmitters. Indeed, we found down-regulation of mRNA levels encoding ChAT, the enzyme synthesizing acetylcholine, which hints at a reduced intrastriatal cholinergic tone. Also, *spf* mice suffering from congenital hyperammonemia due to X-linked deficiency of the urea cycle enzyme OTC exhibit reduced activity of striatal ChAT and increased densities of muscarinic receptors^49^,^50^,^51^. These changes in neurotransmitter systems were accompanied by a significant loss of striatal principal neurons in *spf* mice^52^. Although the altered expression of striatal beta-actin mRNA in LGS-ko mice revealed by our study implies considerable structural alterations in the striatum, it is hardly due to a change in striatal cell numbers, as the expression of other housekeeping genes including SDHA, Hsp90, or B2m was comparable between genotypes. It can rather be expected that the regulation of beta-actin is associated with the reorganization of the cytoskeleton in dendritic spines, which are involved in changes in synaptic plasticity^53^. In hepatocytes, the transcription level of beta-actin is regulated by osmolarity and glutamine, so that hypo-osmolarity and glutamine increase whereas hyper-osmolarity decreases beta-actin mRNA^54^,^55^. Future studies may clarify the role of osmolarity and glutamine synthesis for altered beta-actin expression in the striatum but not the hippocampus of LGS-ko mice suffering from inborn systemic hyperammonemia.

Striatal cholinergic interneurons exhibit tonic firing patterns, which are controlled by D2R signaling stimulated by dopaminergic afferents from the substantia nigra^56^,^57^. They are able to direct corticostriatal synaptic plasticity in such a way that a decrease in cholinergic tone promotes the induction of HFS-LTD^58^, whereas the knockout or pharmacological inhibition of D2R promotes HFS-LTP^59^. The loss of this reciprocal modulation between dopaminergic inputs and intrinsic cholinergic activity in the striatum is thought to cause motor and cognitive disturbances^60^,^61^,^62^. Here, inhibiting D2Rs facilitated LTP induction as expected, but it was also able to balance the difference between LGS-ko and wt mice in HFS outcome. Indeed, under these conditions the magnitude of corticostriatal HFS-LTP was even higher in LGS-ko mice than in wild type, which may be attributed to overexpression of D1R priming AMPAR for synaptic insertion^63^,^64^.

Our study demonstrates a distinct pattern of alterations in properties of long-term depression in the two neuronal circuitries studied. Whereas hippocampal LTD induced pharmacologically by application of either NMDA or the group I mGluR agonist DHPG was not changed at all, a reversal of NMDA-induced LTD and a significant decrease in the magnitude of LTD induced by DHPG was found in the striatum of LGS-ko mice. Similar modifications of chemically-induced LTD were previously observed in an *in vitro* model of hyperammonemia with long preincubation of corticostriatal slices with ammonium chloride^65^,^66^. Resistance of hippocampal NMDA-LTD to high ammonia conditions is in line with previous data in another *in vitro* model of hyperammonemia^43^: a brief pulse of NMDA had readily induced chemical LTD in dissociated hippocampal cultures at DIV14–16 independent of prior ammonium chloride or sham treatment for 36 h.

Opposite long-term alterations in corticostriatal neurotransmission were observed in LGS-ko and wt mice after brief exposure to NMDA. Bath application of NMDA can stimulate both synaptic and extrasynaptic NMDAR which in adult striatal medium spiny neurons are made up largely of GluN2A and GluN2B subunits^67^ with the former prevailing in the composition of synaptic and the latters in extrasynaptic NMDAR^68^. The initial NMDA-induced depression of corticostriatal field responses was shown to result exclusively from activation of NR2A-containing NMDAR^69^. The similarity of its characteristics in LGS-ko and wt mice fits well with the absence of transcriptional alterations in the expression of this subunits. Considering complex functional interactions of synaptic NMDAR and D1R^70^, the reversal of long-lasting NMDA effects to LTP in LGS-ko striatum might be associated with the increased expression of D1R. In the hippocampus, NMDA-LTD could be reversed by concomitant activation of D1R with the specific agonist^71^. It remains to be determined if and how D1R is involved in the mechanisms of long-term effects of NMDA exposure in the striatum.

By contrast to chemical LTD, HFS-induced LTD in the striatum did not differ from wild type. At corticostriatal synapses, LTD elicited by HFS requires the co-activation of D2Rs and group I mGluRs, which stimulate postsynaptic endocannabinoid (eCB) synthesis to persistently reduce glutamate release via the presynaptic eCB receptor CB1R^72^. Moreover, activation of the nitric oxide (NO)-cGMP-PKG signaling pathway is crucial^73^. Chemical LTD evoked in the dorsal striatum by brief activation of group I mGluRs shares major mechanistic requirements with HFS-induced LTD including the activation of group I mGluRs, CB1Rs, and NO synthase^74^, but D2R co-activation is not necessary^72^. However, this may not explain its differential suppression in LGS-ko mice. D2R signaling should be fully preserved in those mice, as the D2R inhibitor sulpiride prevented the occurrence of LTD by HFS in LGS-ko and wt animals to similar extent. Moreover, the absence of genotype-dependent transcriptional changes in the expression of group I mGluRs, D2R, and CB1R genes may imply that the observed alterations in synaptic plasticity are caused downstream of these receptors. As induction of corticostriatal LTD by DHPG requires NO-dependent synthesis of cGMP, decreased activity of NOS or lower efficiency of the NO-cGMP- protein kinase G (PKG) signaling cascade could explain reduced expression of DHPG-LTD in the LGS-ko striatum. For LTD induced by DHPG, the NO-cGMP-PKG signaling cascade is supposed to be located in presynaptic terminals of the corticostriatal projection^74^, whereas PKG is activated in the postsynaptic cells when LTD is induced by HFS^73^. The difference in genotype-dependence of DHPG-LTD and HFS-LTD may therefore be attributed to different sensitivity of pre- or postsynaptic regulatory mechanisms activated by NO. In this regard, it is interesting to note that decreased activity of both hippocampal and striatal NOS and reduced NO release have been previously reported for the *spf* mouse model of congenital hyperammonemia^21^.

In conclusion, exploiting a novel mouse model of congenital hyperammonemia^22^, we showed how a selective increase in blood ammonia without any obvious adaptive changes in amino acid and energy metabolism affects exploratory behavior and synaptic function in related brain regions of adult mouse. Mice lacking hepatic GS showed decreased exploratory activity in a novel environment, which was accompanied by a complex pattern of impaired plasticity in hippocampal and corticostriatal neurotransmission and a deregulation of gene expression. The study allows for the first time to specifically deduce the behavioral and synaptic abnormalities to a chronic increase in blood ammonia and may therefore guide developing neuroprotective strategies.

## Methods

### Animals

Male liver-specific glutamine synthetase (GS) knockout mice (LGS-ko, n = 17) and their wild type littermates (wt, n = 19) at the age of two to four months were used. LGS-ko mice were generated, bred, and genotyped as described previously^22^. Mice were kept on a 12 h day – 12 h night light schedule with *ad libitum* access to food and water. All procedures were in compliance with German law and were approved by the local authorities of the University of Düsseldorf.

### Behavioral testing

In order to examine novelty-induced exploratory behavior and habituation, animals were given a single 20 min trial in a SmartCage^TM^ system^75^ without prior exposure to the apparatus. The testing room was dimly illuminated with indirect white lighting. Mice were placed in the inner space (25 × 16 × 14 cm) of a transparent plastic box equipped with an infrared (IR) processor, instrument amplifyer, motor control, and microcontroller units assembled in its walls and removable floor. The box was WiFi-connected with the host computer that acquired the data using the CageCenter™ program of the SmartCage™ system. Data were analyzed using the Windows-based program CageScore™ (AfaSci, Inc. Burlingame, CA, USA). Activity variables, including activity counts (counts of breaks in x-, y- and z-axis beams), activity time, locomotions (travelled distance and speed) and rearings, were calculated in time blocks of 5 min each.

### Slice electrophysiology

Fourty – 60 min after behavioral testing, animals were sacrificed and brains were immersed in ice-cold modified artificial cerebral spinal fluid (aCSF) with a complete sucrose substitution for NaCl. Horizontal brain slices (400 mm) were cut with a vibratome (Campden Instruments, UK); corticostriatal or hippocampal slice preparations were dissected and one half of the slices designed for electrophysiological recording was collected in a store glass with standard aCSF (in mM: 125 NaCl, 1.8 KCl, 1.2 KH_2_PO_4_, 2.4 CaCl_2_, 1.2 MgCl_2_, 26 NaHCO_3,_ and 10 D-glucose, constantly saturated with 95% O_2_ / 5% CO_2_ gas mixture, pH 7.4), while the other half was processed for mRNA isolation. After at least 2 h preincubation at room temperature in standard aCSF, a single slice was transferred to a submersion-type recording chamber, which was continuously perfused with standard aCSF at a flow rate of 1.5–2 ml/min at 32 °C.

Hippocampal CA1 field EPSPs and corticostriatal field potentials were evoked by stimulation of corresponding synaptic inputs through bipolar nickel-chrome electrodes and recorded with low-resistance (2–4 MΩ) aCSF-filled micropipettes: in hippocampal slices, the stimulating electrodes were placed on the stratum radiatum at the border between CA2 and CA1; in corticostriatal slices, the subcortical white matter at the border between cortex and striatum was stimulated. After the initial testing of stimulus – response relationships at a stimulation frequency of 0.1 Hz, the stimulus intensity was adjusted to induce a field response of approximately 50% of its maximal amplitude and the stimulation frequency was set to 0.033 Hz. Each experiment included a 15–20 min period of control recording, application of high-frequency electrical stimulation (HFS) or chemical stimulus, and 60–90 min monitoring of post-stimulus alterations in responsiveness. HFS consisted of 100 stimuli at 100 Hz repeated 3 times with 30 s intervals in the hippocampal slices and of 4 repetitions with 10 s intervals in corticostriatal slices. The chemical stimuli were short perfusions with 20 μM N-methyl-D-aspartate (NMDA) or 100 μM S-3,5-dihydroxyphenylglycine (DHPG). NMDA and DHPG were purchased from Abcam (Germany), prepared as stock solutions, stored in aliquots at −20 °C, and were thawed and diluted in aCSF immediately before application.

Signals were amplified, digitized at 10 kHz, stored on a hard disk of a PC using Clampex software of pClamp (Axon Instruments), and analyzed off-line, using Clampfit and Excel. Ten consecutive field responses (5 min recordings) were averaged, the slope of averaged hippocampal fEPSPs was measured by straight line fitting, the amplitude of corticostriatal postsynaptic peak response (N_2_ peak^76^,^77^ was measured as an average of its minimum (from the early positivity to the peak negativity) and maximum (from the peak negativity to the late positivity) values. All values were normalized to baseline (the mean values for a 15–20 min control period) and plotted against time.

### Real-time RT-PCR

Striatal and CA1 hippocampal tissues were isolated from 1–3 horizontal brain slices and total cellular mRNA was extracted using a mRNA isolation kit (Quickprep Micro mRNA Purification Kit, GE Healthcare, GB). Real-time RT-PCR was used to detect genotype-related alterations in gene expression. We analyzed the expression of genes encoding receptors for neurotransmitters, known to be involved in hippocampal and corticostriatal plasticity (glutamate, dopamine, acetylcholine, endocannabinoids) and other plasticity-related proteins. Detailed description of the applied protocol was presented previously^25^. The relative mRNA level encoding each gene was estimated by the 2^−ΔΔCt^ method described previously^25^,^78^, where ΔCt = Ct target gene – Ct Rpl13a (ribosomal protein L13a). Rpl13a was selected as the most appropriate housekeeping gene using criteria described previously^79^,^80^. We normalized our reactions on the expression of ribosomal protein L13A (Rpl13a) using the following primers: up: 5′-ATG ACA AGA AAA AGC GGA TG-3′and lo: 5′-CTT TTC TGC CTG TTT CCG TA-3′^81^. Primers for the succinate dehydrogenase complex subunit A (SDHA) were the same as in^22^. Primers for the subunits of AMPA-subtype glutamate receptors were the same as in^43^. Average 2^−ΔΔCt^ values for the WT genotype were set 100% and the individual values for LGS-ko tissues were expressed relative to wt control.

### Statistical analysis

Data are presented as mean ± SEM. Statistical analysis was performed using Excel and GraphPad Prism5 software as indicated. Experiments were repeated in n = number of slices from at least N = 4 animals.

## Additional Information

**How to cite this article**: Chepkova, A. N. *et al*. Impaired novelty acquisition and synaptic plasticity in congenital hyperammonemia caused by hepatic glutamine synthetase deficiency. *Sci. Rep.* **7**, 40190; doi: 10.1038/srep40190 (2017).

**Publisher's note:** Springer Nature remains neutral with regard to jurisdictional claims in published maps and institutional affiliations.

## Figures and Tables

**Table 1:** Alterations in striatal and hippocampal gene expression in mice lacking liver-specific GS (LGS-ko).

Transcript	Hippocampus (CA1)	Striatum
Neurotransmitters and neuromodulators		
GluA1	135 ± 15	110 ± 16
GluA2	116 ± 20	96 ± 15
GluA3	111 ± 21	81 ± 19
GluN1	109 ± 8	108 ± 10
GluN2A	112 ± 13	106 ± 18
GluN2B	65 ± 8^#^	131 ± 15
GluN2C	120 ± 17	132 ± 19
GluN3A	92 ± 11	110 ± 16
mGluR1	175 ± 27^#^	123 ± 28
mGluR5	128 ± 29	137 ± 27
M1R	85 ± 9	136 ± 11*
D1R	157 ± 47	146 ± 11***
D2R	108 ± 24	130 ± 16
CB1	106 ± 15	160 ± 33
A1R	73 ± 11^#^	80 ± 15
Plasticity-related enzymes		
ChAT	100 ± 16	63 ± 7*
nNOS	98 ± 11	102 ± 12
eNOS	126 ± 18	140 ± 21
Structural proteins and Housekeeping genes		
PSD 95	137 ± 16	121 ± 21
Beta-actin	96 ± 20	211 ± 18**
SDHA	100 ± 18	110 ± 19
GAPDH	165 ± 17	120 ± 15
B2m	292 ± 63*	126 ± 21
Hsp90	284 ± 64*	130 ± 18

Gene expression in LGS-ko brain tissues is presented in percent to corresponding wild type values. Statistical significance was assessed by the two-tailed (marked by asterisks, *p < 0.05; **p < 0.01, ***p < 0.001) or one-tailed (marked by hash signs ^#^p < 0.05) Mann-Whitney *U*-test.
